# Non-stick syringe needles: Beneficial effects of thin film metallic glass coating

**DOI:** 10.1038/srep31847

**Published:** 2016-08-30

**Authors:** Jinn P. Chu, Chia-Chi Yu, Yusuke Tanatsugu, Mikito Yasuzawa, Yu-Lin Shen

**Affiliations:** 1 Department of Materials Science and Engineering, National Taiwan University of Science and Technology, Taipei 10607, Taiwan; 2 Department of Chemical Science and Technology, Tokushima University, Tokushima 770-8506, Japan; 3 Department of Mechanical Engineering, University of New Mexico, Albuquerque, NM 87131, USA

## Abstract

This paper reports on the use of Zr-based (Zr_53_Cu_33_Al_9_Ta_5_) thin film metallic glass (TFMG) for the coating of syringe needles and compares the results with those obtained using titanium nitride and pure titanium coatings. TFMG coatings were shown to reduce insertion forces by ∼66% and retraction forces by ∼72%, when tested using polyurethane rubber block. The benefits of TFMG-coated needles were also observed when tested using muscle tissue from pigs. In nano-scratch tests, the TFMG coatings achieved a coefficient of friction (COF) of just ∼0.05, which is about one order of magnitude lower than those of other coatings. Finite-element modeling also indicates a significant reduction in injection and retraction forces. The COF can be attributed to the absence of grain boundaries in the TFMG coating as well as a smooth surface morphology and low surface free energy.

Syringe needles are common medical devices used in the transdermal delivery of drugs. The interaction between the needle and tissue greatly influences the discomfort associated with the use of these devices^1^,^2^. The force necessary for penetration can be affected by many factors, such as the geometry of the point, the method of insertion, and characteristics of the tissue. Fine needles with a smaller gauge size are becoming increasingly popular for their ability to reduce the pain associated with needle insertion. Insulin needles and dental needles are examples of fine needles used for individual and clinical use, respectively^3^,^4^. Few studies have reported on the coatings used in the preparation of needles for medical applications, despite their importance on the performance of the devices. The surface of most medical needles is coated with silicone fluid lubricants to reduce friction and thereby reduce the amount of force required for insertion. However, the effectiveness of these coatings decreases with use, particularly when used for the sewing of skin.

Diamond-like carbon (DLC) coatings have been applied in biomedical applications, to take advantage of their exceptionally low friction properties and biocompatibility^5^. DLC coatings on the needles used in ophthalmic surgery have been shown to reduce the friction involved in penetrating the cornea of pig eyes by about 30%^6^. However, DLC is brittle and has high internal compressive stress, leading to the poor adhesion to the metallic substrates^7^. A new class of metallic glass (MG) exhibit high strength, elasticity, and good corrosion and wear resistance due to a lack of long-range atomic periodicity^8^,^9^. This makes MGs a promising alternative candidate for biomedical applications^10^. Researchers have recently reported that MGs exhibit excellent biodegradability and biocompatibility^11^,^12^,^13^. In previous studies, thin film metallic glass (TFMG) has been shown to provide effective antibacterial properties^14^,^15^ as well as sharpness and durability when applied to surgical blades^14^,^16^. In this study, we applied TFMG coating to 304 stainless-steel (SS304) dental needles (size 31 G) and compared the results with conventional crystalline metallic coating, pure titanium (Ti), and ceramic coating (titanium nitride, TiN) with regard to the surface properties of the devices. We also used numerical simulation to elucidate the influence of COF on the performance of the needles.

## Results and Discussion

**Figure 1:**
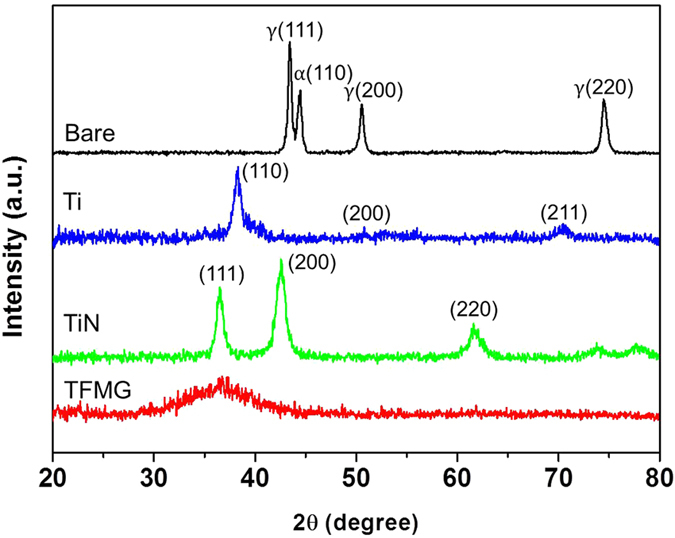
XRD patterns obtained from bare needle and needles with TFMG, Ti, and TiN coatings.

The glass transformation temperature (*T*_*g*_) and crystallization temperature (*T*_*X*_) of Zr_53_Cu_33_Al_9_Ta_5_ TFMG were measured at 467.9 and 519.8 °C, respectively. Figure 1 presents the XRD patterns from bare, TFMG, Ti, and TiN films. In the XRD patterns, a broad hump in the 2 theta range from 30 to 50° indicates the amorphous structure of TFMG without obvious crystalline Bragg peaks. The diffraction patterns of Ti revealed three characteristic Bragg peaks associated with orientations of (110), (200), and (211), according to JCPDS#89-4913 standards. Strong peaks were detected at 36.6° and 42.5° for (111) and (200) planes in the TiN (JCPDS#65-5759). Three strong Bragg peaks for (111), (200) and (220)γ-Fe (austenite) and one peak for (100)α-Fe (ferrite) were detected in the bare samples.

**Figure 2:**
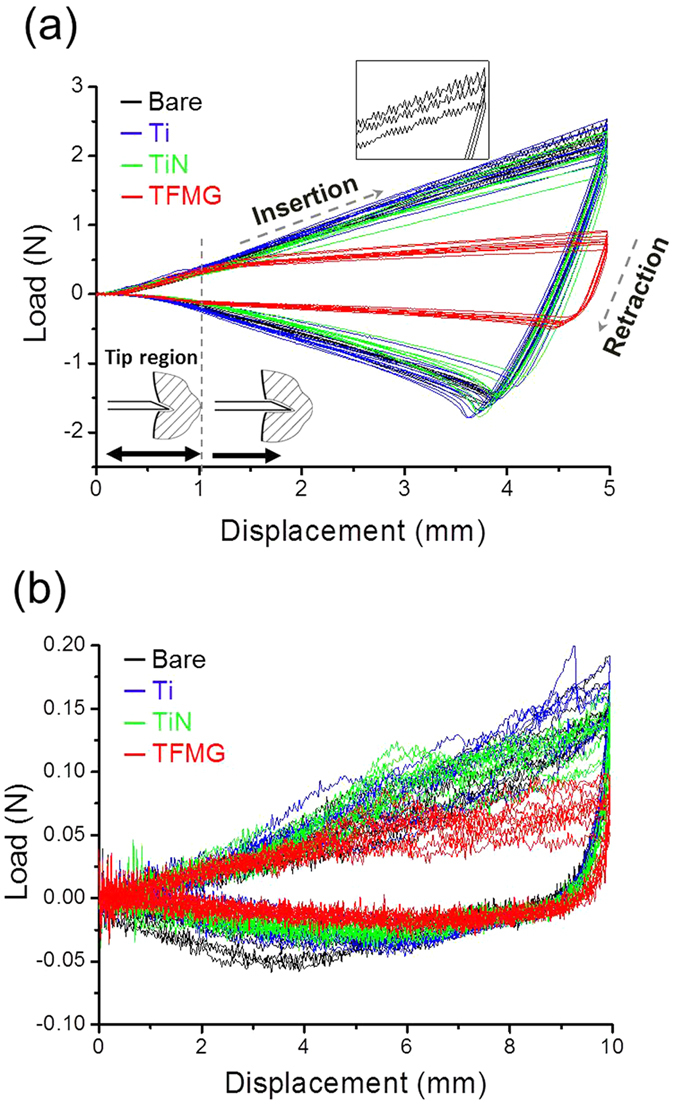
Load-displacement curves obtained from bare needle and needles with TFMG, Ti, and TiN coatings upon insertion and retraction into (**a**) PU rubber (**b**) pork muscle. Inset in (a) is 2x enlargement of the curves, revealing serrated (jerky) curves obtained from three bare needles under nearly maximal loads.

Figure 2 presents the insertion/retraction force measurement results in rubber and pig muscle. Insertion force is due mainly to the effects of cutting and friction, whereas retraction force is due primarily to friction. Deformation of the tip of the needle upon coming into contact with the specimen would result in erroneous friction results; however, no tip damage was observed before or after any of the insertion/retraction tests. As indicated by the load vs. displacement curve in Fig. 2(a), (using PU), the forces associated with the insertion/retraction of TFMG-coated needles was far below that of the other specimens. Nonetheless, the forces of all specimens were nearly the same for the first 1 mm of insertion due to the fact that the total length of the tapered needle point is approximately 1 mm. Puncture force depends almost entirely on needle tip geometry^17^, which is nearly the same for all specimens in this study. The contact area between the needle and rubber increased as the needle advanced into the PU block; therefore, friction became increasingly dominant over the cutting force induced by the tip of the needle. The curves obtained from TFMG increased only slightly, whereas bare, Ti and TiN-coated samples presented dramatic increases with displacement. The inset in Fig. 2(a) presents a serrated (jerky) curve for the bare sample under high load, which is the result of stick-slip motion associated the high friction surfaces. In contrast, the curve for TFMG is smooth. Table 1 lists the peak forces associated with the insertion and retraction of needles for the various coating materials. The insertion and retraction forces associated with TFMG-coated samples are ∼66% and ∼72% lower than those of the bare samples. We observed a pronounced increase in the force associated with displacement for all needles except those with TFMG coatings.

**Figure 3:**
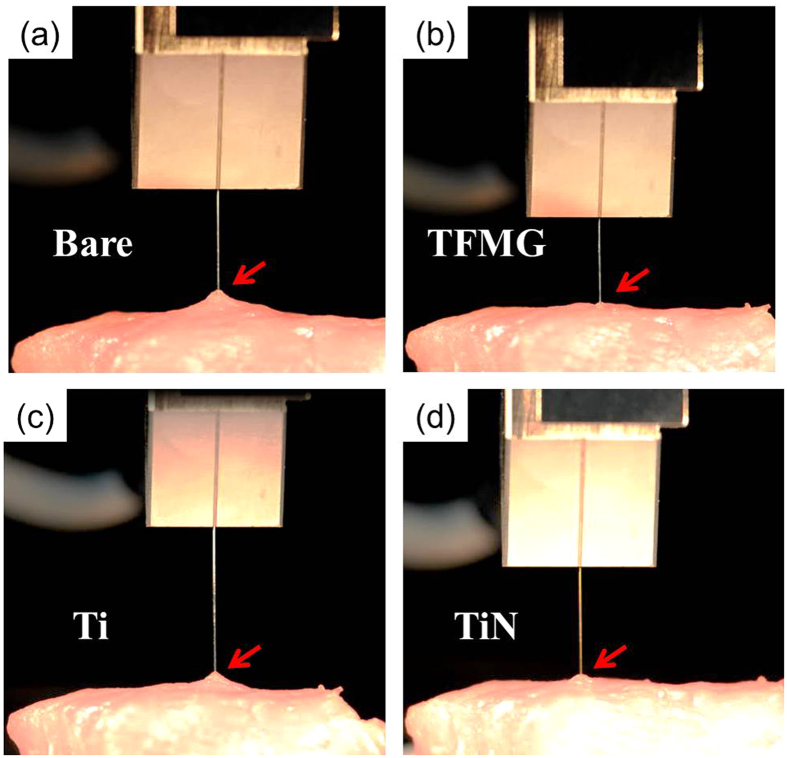
Photographs of (**a**) bare, (**b**) TFMG-coated, (**c**) Ti-coated and (**d**) TiN-coated needles during retraction from pork muscle. Arrows indicate signs of needles sticking to pork muscle in (**a**), (**c**) and (**d**), but not in (**b**).

This study used pig muscle as a phantom material to elucidate the effects of coatings on biomaterials. Load vs. displacement curves obtained using pig muscle [Fig. 2(b)] tended to scatter, due to the inhomogeneous nature of muscle comprising muscle tissue, blood vessels, and nerves. Despite the difference in hardness of PU and pig muscle, the force measurement curves presented the same trends, as shown in Table 1. The insertion force of TFMG-coated samples was ∼47% lower than that of bare samples, whereas the retraction force was ∼41% lower. Frames obtained from videos of needle retraction (Fig. 3), illustrate the effects of coatings on the sticking behavior between the needle and muscle tissue. (The full video can be found in Supplementary Video S1) As shown in Fig. 3(a), muscle tissue clearly stuck to the bare needle during retraction. The area of elevated tissue in the image is referred to as a hump. The lack of an observable hump in the image of TFMG-coated needle is a clear illustration of non-stick characteristics of TFMG [Fig. 3(b)]. In comparison, the bare and Ti-coated sample presents a pronounced hump and the TiN-coated needles present a small hump.

**Figure 4:**
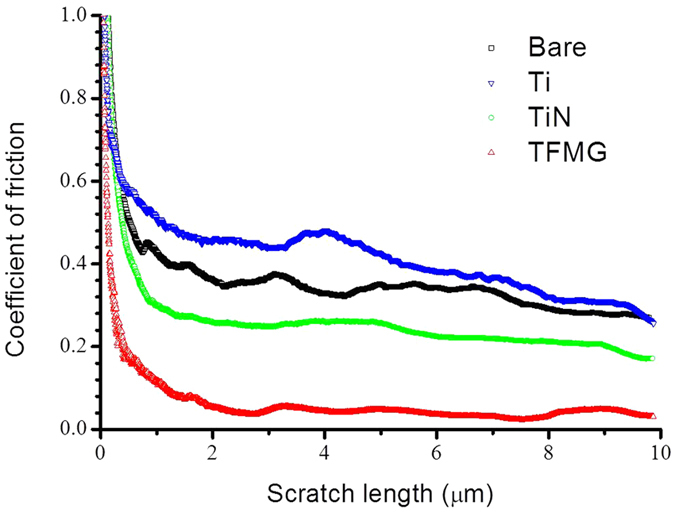
Coefficient of friction (COF) vs. scratch distance obtained from bare needle and needles with TFMG, Ti, and TiN coatings.

Nano-scratch testing was used to measure the COF of the needle specimens in order to identify the factors influencing force measurements (i.e., the stickiness of the needles). COF values were obtained by dividing the measured lateral force by the applied normal force while scratching the surface of the coating. These results were then plotted against scratch length, as shown in Fig. 4. The high initial COF values (particularly those in the low load region), are associated with the settling of the indenter head. After leveling off, the average COF was calculated from scratch lengths of between 2 and 10 μm, as shown in Table 1. The average COF value obtained from samples with a TFMG coating was 0.05, which is approximately one order of magnitude lower than those of conventional coatings such as TiN (0.23) and Ti (0.39). The uniformity in the load vs. displacement curves of TFMG can be attributed primarily to low COF values.

Tribological properties are not intrinsic; therefore, investigations must be conducted under a wide range of conditions. BMG has been shown to outperform conventional structural materials in terms of friction characteristics^18^,^19^; however, the mechanism dominating these effects has yet to be elucidated. In this study, we examined the effects of surface coatings on COF from the perspectives of microstructure, surface roughness and wettability. Balasubramaniam *et al*.^20^ investigated the effects of grain size on the tribological behavior of a nanocrystalline nickel film prepared by electrodeposition. Pure nickel (grain size of 61 μm) exhibited a high COF of approximately 0.60, whereas the COF for nanocrystalline nickel (grain size of 8 nm) was only 0.16. In this study, we used Scherrer’s formula^21^ in conjunction with XRD results to determine the size of grains in our coatings, as follows:


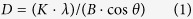


where *D* is the average grain size; *K* is a dimensionless shape factor (*K* = 0.89); *B* is the full width at half maximum (FWHM) of the diffraction-peak; *λ* is X-ray wavelength (the *λ*_Kα_ of Cu is 0.154 nm); and θ is the Bragg angle of the major diffraction peak. The average size of grains were as follows: bare needle (29 nm), Ti (13.4 nm), and TiN (9.8 nm). The smaller grain size of TiN resulted in COF values lower than those of Ti and the bare needle. The low COF value of TFMG can also be attributed to the absence of grain boundaries due to its amorphous structure. Coating samples with TiN resulted in a 36% reduction in COF (from 0.36 to 0.23); however, this led to only slight decreases in insertion and retraction forces.

Surface wettability also plays an important role in the tribological properties of materials. COF against human skin has been shown to decrease with an increase in the hydrophobicity of the sliding material^22^. Skin and muscle tissue contain a large fraction of fluids, which can have a considerable effect on resistance to the insertion of a needle. In the investigation of hydrophobicity, we determined the SFE of bare SS304, TFMG, Ti, and TiN coatings using CA measurements. The surface roughness was shown to be less than 5 nm; i.e., far below the level of significance of 500 nm^23^. We employed the Owens–Wendt method^24^, consisting of two components of dispersion and polar SFE, as follows:


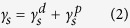


where *γ*_*s*_ is the SFE, *γ*_*s*_^*d*^ is the dispersion component of SFE, and *γ*_*s*_^*p*^ is the polar component of SFE. We used bipolar (DI-water) and dispersive liquids (diiodomethane) to determine the SFE of the specimens in this study. Table 2 presents the water CA, diiodomethane CA, *γ*_*s*_^*d*^, *γ*_*s*_^*p*^, and *γ*_*s*_ of the samples in this study, revealing the slight hydrophobicity of TFMG, TiN and Ti, with angles of 104.0°, 91.7°, and 93.3°, respectively. In comparison, the bare needles presented hydrophilicity of 82.1°. The SFE values were as follows: TFMG (23.0 mN/m), and bare (37.6 mN/m), Ti (34.4 mN/m), and TiN (36.0 mN/m). The dispersive component, *γ*_*s*_^*d*^, made the greatest contribution to SFE in all of the samples. Similarities in the insertion and retraction forces of Ti-coated and bare needles can be attributed to similarities in the values of COF and SFE. The slightly lower insertion and retraction forces of TiN-coated needles may be attributed to higher SFE, despite the fact that the COF is far below those of Ti-coated and bare needles. The excellent performance of the TFMG can be attributed to the combined effects of smooth surface, low SFE and COF values, and amorphous structure.

**Figure 5:**
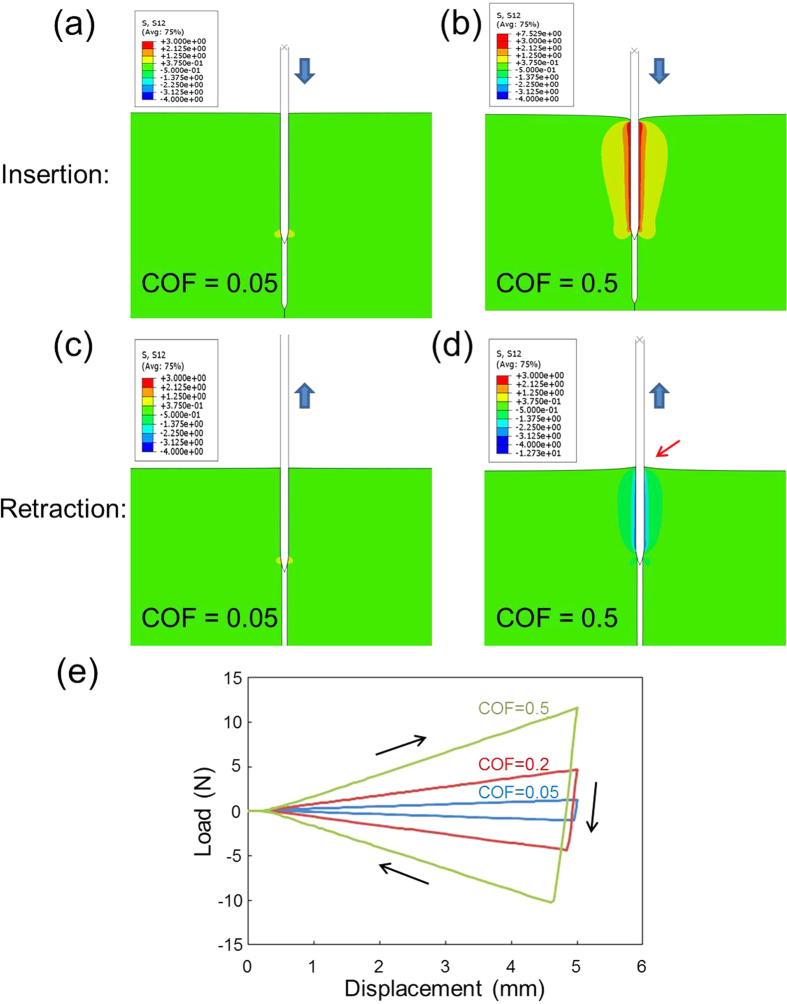
Contour plots of shear stress (*σ*_*12*_) developed in PU rubber under insertion of needles into PU rubber: (**a**) COF = 0.05 and (**b**) COF = 0.5; retraction of needles from PU rubber: (**c**) COF = 0.05 and (**d**) COF = 0.5.

We also conducted FEM simulations to provide further insight into the mechanisms involved in the friction of needles. Figure 5(a,b) are contour plots of shear stress (*σ*_*12*_) that developed when needles (COF of 0.05 and 0.5), were inserted into PU rubber, whereas Fig. 5(c,d) plot the effects of retraction. An intensive stress field of *σ*_*12*_ appeared at the interface of the rubber and needle (COF of 0.5) during injection and retraction. This can be attributed directly to frictional forces. The effects of rubber sticking to the needle were also observed during retraction, as indicated by the red arrow in Fig. 5(d). Figure 5(e) presents the load-displacement curves associated with the insertion and retraction of needles with COF values of 0.05, 0.2, and 0.5. The simulated load-displacement curves resemble those of the friction-dominant part from the measurements shown in Fig. 2(a). During insertion frictional slide in conjunction with the squeezing action occurs. At the initial stage of retraction, the peak pushing force (positive) rapidly changes to the peak pulling force (negative), beyond which frictional slide and elastic recovery of the PU rubber ensue. The peak load in Fig. 5(e) was shown to drop noticeably with a decrease in COF value during both insertion and retraction, thereby indicating a high degree of correlation between the results of FEM and those obtained in experiments. Low insertion/retraction forces and a lack of adhesion to TFMG-coated needles could be expected to reduce the pain associated with the use of needles in a clinical setting.

In summary, this study investigated the performance of syringe needles coated with TFMG, Ti, and TiN. In experiments involving PU rubber blocks and pig muscles, TFMG-coated needles were shown to significantly outperform bare needles as well as those coated with Ti and TiN with regard to insertion and retraction forces. The beneficial effects of TFMG coatings can be attributed to low COF (∼0.05) and low SFE (∼23 mN/m) values, associated with a smooth surface morphology and grain boundary-free structure.

## Methods

Prior to film deposition, the needles were cleaned to remove surface contamination. TFMG, titanium (Ti), or titanium nitride (TiN) films were grown on dental needles and Si(001) wafers to a thickness of 80 nm using radio-frequency magnetron sputtering at a base pressure <1 × 10^−6^ Torr. TFMG and Ti coatings were respectively deposited using a Zr_53_Cu_33_Al_9_Ta_5_ (at%) alloy target or pure Ti (99.95 wt%) target with no intentional heating at an Ar pressure of 3 mTorr. TiN coatings were prepared using a pure Ti target in mixed Ar:N_2_ (2:3) atmosphere with an applied substrate bias of −50 V. The needles were rotated during deposition to ensure uniform coating on all surfaces. A typical amorphous structure is observed in TFMG and the adhesion between film and substrate is good, as shown in a cross-sectional transmission electron micrograph in (Supplementary Fig. S1).

The thermal properties of the TFMG were characterized using differential scanning calorimetry (DSC, Netzsch 404 F3 Pegasus) at temperatures of up to 600 °C at a heating rate of 40 K/min under Ar atmosphere. The crystallographic structures were characterized using low-glancing-angle X-ray diffractometer (XRD, D8 Discover) with Cu *K*_*α*_ radiation at 40 kV and 200 mA.

The insertion and retraction forces were measured at speeds of 5 mm per minute using polyurethane (PU) rubber and pig muscle using a 5 N-load cell mounted on a material testing system (MTS 42.503). (The experiment setup is illustrated in Supplementary Fig. S2). Cubes of pig muscle were obtained from the hindquarters of pigs, which was purchased from a supermarket and inspected by the Certified Agricultural Standards (CAS) in Taiwan. The insertion and retraction experiments using pork muscles were conducted within 30 minutes after removal from the refrigerator to ensure freshness. Each coating was tested using at least ten needle samples. The nano-tribological properties of the needles were determined using nano-scratch tests at room temperature under a normal ramping load of up to 1000 μN using a TI 950 TriboIndenter. The relative humidity was controlled in 50% ± 2%. A conical nanoindentation tip was used to measure the coefficient of friction (COF) of all test samples. For the nano-scratch test, a scratch speed of 0.33 μm/s is used over a total length of 10 μm. There are at least 5 scratches per sample under ramping loads from 0 to 1 mN. The surface wettability and surface free energy (SFE) of bare SS304 sheets and coated Si(001) wafers was evaluated using the sessile drop contact angle method with polar (deionized water) and dispersive (diiodomethane) liquids using a contact angle goniometer (Sindatek Model 100SB). The droplet volume was 2 μL and the calculation of surface free energy (SFE) was based on contact angle (CA) measurements in accordance with the Owens-Wendt theory^24^.

Finite element modeling (FEM) was used to investigate the effect of COF on the mechanical behavior of the needle and target material during insertion and retraction (see Supplementary Fig. S3). The insertion/retraction characteristics were based on COF values (0.05-0.5) using a needle with diameter of 0.25 mm as a rigid body. The PU material used to test insertion characteristics was assumed to possess linear elasticity with Young’s modulus of 25 MPa and Poisson’s ratio of 0.45, following the general range of its reported elastic property^25^. A pre-existing long hole with a diameter of 0.16 mm was built into the model. The pre-existing hole enables the study of frictional effect and avoids the uncertainty involved in the simulation of puncture. Although the sizes of needle and insertion hole, along with the elastic property of the target material, can affect the numerical results, the current model is able to provide quantitative insight into how contact friction dictates the penetration force and overall deformation behavior. The insertion distance was 5 mm using the same experiment setup outlined above.

## Additional Information

**How to cite this article**: Chu, J. P. *et al*. Non-stick syringe needles: Beneficial effects of thin film metallic glass coating. *Sci. Rep.* **6**, 31847; doi: 10.1038/srep31847 (2016).

## Supplementary Material

Supplementary Information

Supplementary Video S1

## Figures and Tables

**Table 1:** Maximum forces generated upon insertion and retraction of needles as well as surface roughness and COF of bare, TFMG-, Ti- and TiN-coated needles.

Coating material	Insertion peak force (N)		Retraction peak force (N)		Surface roughness, R_q_ (nm)	Coefficient of friction
	Rubber	Pork muscle	Rubber	Pork muscle		
Bare	2.27 ± 0.13	0.15 ± 0.02	1.51 ± 0.05	0.044 ± 0.010	16.2 ± 2.7	0.33 ± 0.03
Ti	2.27 ± 0.18	0.16 ± 0.02	1.64 ± 0.12	0.039 ± 0.006	11.9 ± 1.6	0.36 ± 0.03
TiN	2.06 ± 0.24	0.14 ± 0.02	1.50 ± 0.20	0.034 ± 0.004	14.2 ± 0.7	0.23 ± 0.01
TFMG	0.78 ± 0.09	0.08 ± 0.01	0.43 ± 0.05	0.026 ± 0.003	10.0 ± 1.7	0.05 ± 0.01

**Table 2:** Contact angle and surface free energy measurement results of bare SS304, Ti, TiN and TFMG coatings.

Coating material	Water (°) CA	Diiodo-methane CA (°)	Dispersive component, 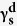	Polar component, 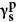	Surface free energy, 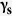 (mN/m)
Bare	82.1 ± 1.8	46.8 ± 1.4	36.0	1.6	37.6
Ti	91.7 ± 0.8	50.5 ± 1.8	34.0	0.4	34.4
TiN	93.3 ± 0.2	47.0 ± 1.2	35.9	0.1	36.0
TFMG	104.0 ± 1.1	69.9 ± 1.3	22.9	0.1	23.0
