# Supplementary material for: Non-stick syringe needles: Beneficial effects of thin film metallic glass coating
Source: Sci Rep. 2016 Aug 30;6:31847. doi: 10.1038/srep31847 (PMC5004157; doi:10.1038/srep31847)
Supplement: Supplementary Information [file srep31847-s1.pdf]

Supplementary Information

Non-stick syringe needles: Beneficial effects of thin film metallic glass coating

Jinn P. Chu, Chia-Chi Yu, Yusuke Tanatsugu, Mikito Yasuzawa, Yu-Lin Shen

Supplementary Video S1. Video file taken during needle insertion and retraction from pork muscles for all needles evaluated in this study. Arrows indicate signs of needles sticking to pork muscle in bare, Ti-coated and TiN-coated needles, but not in TFMG-coated needle.

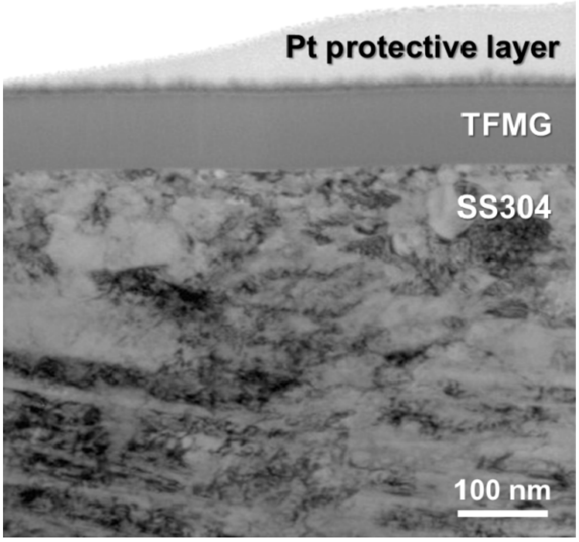

Supplementary Figure S1. Typical cross-sectional transmission electron micrograph of TFMG/SS304 sample. The Pt protective layer was deposited during the sample preparation using a focused ion beam system. The TFMG layer presents a typical amorphous structure with no granular contrast. Adhesion between the film and substrate is good.

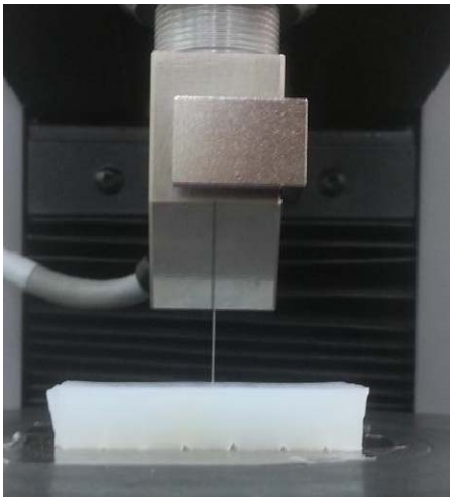

Supplementary Figure S2. Illustration of experiment setup used in force measurement associated with the insertion and retraction of needles into PU rubber.

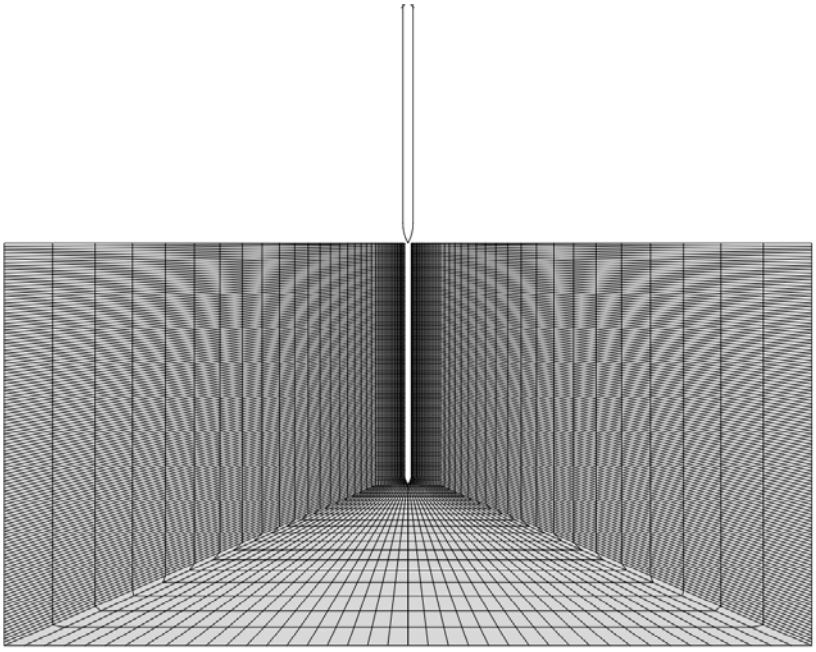

Supplementary Figure S3.

Illustration of force measurements associated with insertion and retraction of needles into PU rubber in numerical model.
